# How many operating rooms are needed to manage non-elective surgical cases? A Monte Carlo simulation study

**DOI:** 10.1186/s12913-015-1148-x

**Published:** 2015-10-28

**Authors:** Joseph M. O’Brien Antognini, Joseph F. Antognini, Vijay Khatri

**Affiliations:** Department of Astronomy, The Ohio State University, Columbus, OH USA; Peri-operative Services and the Department of Anesthesiology and Pain Medicine, University of California, Davis, Sacramento, CA USA; Department of Surgery, University of California, Davis, Sacramento, CA USA

## Abstract

**Background:**

Patients often wait to have urgent or emergency surgery. The number of operating rooms (ORs) needed to minimize waiting time while optimizing resources can be determined using queuing theory and computer simulation. We developed a computer program using Monte Carlo simulation to determine the number of ORs needed to minimize patient wait times while optimizing resources.

**Methods:**

We used patient arrival data and surgical procedure length from our institution, a tertiary-care academic medical center that serves a large diverse population. With ~4800 patients/year requiring non-elective surgery, and mean procedure length 185 min (median 150 min) we determined the number of ORs needed during the day and evening (0600–2200) and during the night (2200–0600) that resulted in acceptable wait times.

**Results:**

Simulation of 4 ORs at day/evening and 3 ORs at night resulted in median wait time = 0 min (mean = 19 min) for emergency cases requiring surgery within 2 h, with wait time at the 95th percentile = 109 min. Median wait time for urgent cases needing surgery within 8–12 h was 34 min (mean = 136 min), with wait time at the 95th percentile = 474 min. The effect of changes in surgical length and volume on wait times was determined with sensitivity analysis.

**Conclusions:**

Monte Carlo simulation can guide decisions on how to balance resources for elective and non-elective surgical procedures.

## Background

Millions of surgical procedures are performed in the United States annually, and many of these are done on an urgent or emergency basis. Consequently, timely access to surgical care is vital to achieve optimal outcomes [1]. Most hospitals devote peri-operative resources (operating rooms, staff, physicians, equipment) to both elective and non-elective surgeries, however, the division of these costly resources depends, in part, on the relative mix of these two classes of cases (elective vs. non-elective). Resource planning for elective surgeries is relatively straightforward, while planning for non-elective surgery is often more challenging. For example, how many operating rooms (ORs) should be devoted to non-elective surgeries?

Queuing theory (also known as waiting-line modeling) and other operational research techniques have been used in a variety of healthcare settings to determine how long patients must wait for care relative to the available resources [2–7]. In this paper, we describe a model that predicts the waiting time for patients needing urgent surgical care. We used standard queuing theory models and Monte Carlo techniques to test the validity of our findings and predictions.

## Methods

The University of California Davis Medical Center (UCDMC) is a 578-bed facility located in Sacramento, California and is part of the University of California Davis Health System. UCDMC has 33 ORs devoted to surgical care, including an outpatient facility (4 ORs), a pediatric facility (5 ORs) and the Pavilion OR area (24 ORs).

The University of California, Davis administrative office of the institutional review board determined that this study was a quality improvement project that did not constitute human research and approved the use of administrative data in this simulation study. Administrative data for surgical procedures performed at UCDMC were used to determine: 1) the “arrival” rate of patients requiring urgent and emergency surgical care in the Pavilion ORs, which was defined as the time when a schedule request was submitted; 2) the length of the surgical procedure (defined as the time the patient wheeled into the OR and the time the patient left the OR). Urgent cases are performed primarily in the Pavilion ORs, although some urgent pediatric cases are performed in the pediatric ORs. In 2013, 22,908 surgical procedures were performed; 75 % of these were elective. The remaining cases were “add-on” elective (5 %) urgent (15 %) and emergency (5 %). For the purposes of this simulation, we excluded urgent and emergency pediatric cases performed in the pediatric unit, as that unit functions somewhat independently. Thus, there were 4802 non-elective cases performed in the Pavilion ORs in 2013: An add-on elective case is defined as one that could wait several days; an urgent case is defined as one that must enter the OR within 24 h, or sooner, depending on the clinical need; urgent cases are further divided into classifications of 0 to 4–6 h (urgent1), 8–12 h (urgent2) and 24 h (urgent3). An emergency case is one that must enter the OR within 2 h. For example, a patient with penetrating trauma and hypotension would be expected to enter the OR within 5–30 min after the decision is made to perform surgery.

The average arrival rate (patients/min) was calculated by dividing the number of patients in each classification by the number of minutes in a year (525,600 min/year). The length of surgery was not normally distributed (it was skewed towards longer procedures times) and was better described using a log normal distribution, consistent with published results [8]. The arrival rate followed a Poisson distribution.

The Monte Carlo Markov chain program was written in the Python language, version 2.6.6 (www.python.org; accessed 6-1-14). Source code of our program is freely available online (https://github.com/joe-antognini/or-wait-times) and we release the code under the Massachusetts Institute of Technology license. The program takes as input: 1) the arrival rate (patients/minute) for each case class; 2) the mean surgical length and standard deviation for each case class (using a log-normal distribution); 3) the set-up and clean-up time (e.g., the pre-operative time spent by the OR staff and anesthesia care team preparing for a case and the post-operative time needed to clean-up the OR and take the patient to the post-anesthesia care unit). This time was set at 60 min (based on our experience at our institution), but was adjusted in some simulations to determine the effect of faster or longer “down” time when the OR staff were not available. Adjusting this time could also reflect changes in operative time. We simulated a 5 year period; data for the initial 2 months was discarded to allow the program time to achieve steady-state.

The program steps through each minute of time and first randomly draws the number of patients in each class who arrive in that minute from Poisson distributions. The arrival time can be thought of as the time when the decision is made to perform surgery and the case is scheduled. Each simulated patient is given a random surgery time drawn from a log-normal distribution. If there are any available ORs, the patients are placed in the ORs starting with the most urgent class. If no ORs are available the patients are placed on a waiting list. When the next OR becomes available the patient in the most urgent class who has been waiting the longest is placed in the OR. Each simulated patient’s class, surgery time, and wait time is recorded. We performed 4–6 simulations (each a 5 year period) in which we changed the number of ORs, the length of surgery/clean-up time or the volume of patients (by adjusting the arrival rate). Using these 4–6 simulations of each set of parameters (number of ORs, surgery/clean-up length, volume) we calculated the means of the mean, standard deviation, median, 95th percentile, and maximum values of wait times. We define the wait time as the time between when the decision is made to perform surgery and when the patient can enter the OR (i.e., the OR is ready to accept the patient). The parameters used (patient arrival rate, mean surgical duration or length and standard deviation of the surgical duration) are shown in Table 1.

**Table 1:** Parameters used to generate wait times

Urgency Class	Mean arrival time	Mean surgery duration	Standard deviation of surgery duration
	(Patients/min)	Natural log	Natural log
Emergent	.001607686	5.00716	0.583642
Urgent 1	.003232496	4.96477	0.677607
Urgent 2	.002665525	5.05842	0.651279
Urgent 3	.000334855	5.00069	0.570812
Add-on	.001288052	5.01655	0.713405

The mean arrival time (patients/minute), mean surgical duration and standard deviation of the surgical duration are shown for each urgency class. The mean surgery durations are expressed as the mean of the natural logarithms of the durations (i.e., each duration was log-transformed and the mean determined). The standard deviations are expressed as the natural logarithms

A second statistical approach using standard bootstrapping techniques was taken to evaluate the uncertainties on the median and 95th percentiles of the wait times. To do this, we took the wait times generated by the Monte Carlo simulation and randomly sampled from this data set with replacement until we had generated a re-sampled data set with as many points as are in the original data set. For example, on each draw from the original sample, any data point is equally likely to be picked as any other, independent of whether that data point had already been picked in a previous draw. Thus, this re-sampled data set contains some data points from the original data set multiple times, and others not at all. The median and 95th percentiles were then calculated for this re-sampled data set. This entire process was then repeated 100 times, producing a distribution of median and 95th percentiles of wait times from the re-sampled data sets. The standard deviation of these distributions was then taken to be the uncertainty of the median and 95th percentile wait times from the original data set.

For comparison purposes, we determined wait times using a multiple server, multiple priorities waiting line model. In this approach, an estimate of mean surgical time must be used. The surgical durations were not normally distributed, i.e., there was rightward skewing of the durations. Using the mean of the data would potentially introduce error because the mean did not represent the central tendency of the data. Therefore, we performed two separate calculations using two means: one calculated from the raw data of surgical times (as noted above) and the second from the log-transformed data (i.e., we took the inverse log of the mean of the log-transformed data). We then used each of these two means to determine average wait times. A comparison of the wait times between the two calculations would provide an estimate of the error of using the mean surgical duration when there is rightward skewing. The program developed by Stevenson and Ozgur [9] has a maximum of four priority classes, so we modified the Monte Carlo simulation model to include only four classes by combining the arrival rates for the 0–24 h class and the add-on elective class.

## Results

**Fig. 1:**
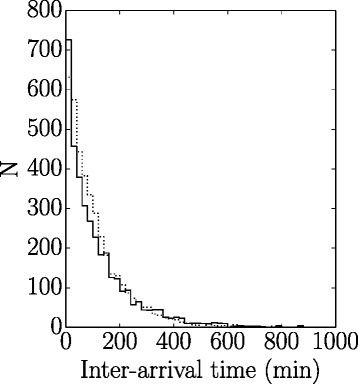
Shown are histograms of patient inter-arrival times (all urgency classes combined); bin width = 20 min. *Solid line*: actual data from University of California Davis Medical Center for a 1 year period. *Dashed line*: simulated data (1 year period). Note the similar distribution of times. The slightly greater peak in the actual data is likely due to two or more patients being scheduled <20 min apart even though the decisions to perform surgery for these patients might have been >20 min apart

The distribution of inter-arrival times are shown in Fig. 1 for real data for 1 year (2013) at UCDMC and for simulated data using the Monte Carlo simulation. Note that in both situations inter-arrival times followed a Poisson distribution.

**Fig. 2:**
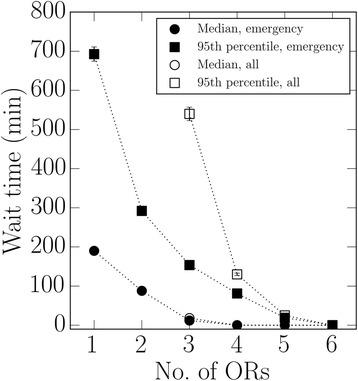
This graph shows wait times (median and 95th percentile) according to the number of operating rooms (ORs) for emergency patients and for all patients combined. Wait times increased exponentially as the number of ORs decreased. The error bars are (±) one standard deviation; unseen error bars are contained within the corresponding symbol. When 1 or 2 ORs were used we show only the wait time for emergency patients because simulations generated surgical demand (total surgical time for all patients) that exceeded capacity which thereby resulted in some simulated urgent patients not being treated

We start with the simplest model in which the number of ORs available during the night is equal to the number of ORs available during the day. Table 2 shows the wait time according to the number of ORs used to service patients. This application of the model assumed that patients would enter the OR when an OR becomes available, regardless of urgency, e.g., an add-on elective patient could receive surgery during late evening or early morning. Increasing the number of available ORs from 3 to 5 decreased utilization from 74.8 to 45 %; mean wait times likewise decreased, reaching just a few minutes for 5 ORs for most urgency classes. For example, when running 4 ORs, wait times at the 95th percentile ranged from 84 min for emergency cases to 256 min for add-on elective cases. When running just 3 ORs, however, the 95th percentile was 155 min (i.e., 5 % of emergency patients would need to wait more than 155 min) (Table 2). Decreasing the number of ORs increased wait times exponentially (Fig. 2).

**Table 2:** Wait times (minutes) according to urgency classification and number of operating rooms

		Number of Operating Rooms		
		3 (*n* = 6)	4 (*n* = 4)	5 (*n* = 4)
Emergent	Mean	39 ± 1	13 ± 1	4 ± 1
	Median	10 ± 2	0 ± 0	0 ± 0
	95th %ile	155 ± 4	84 ± 3	27 ± 1
Urgent1	Mean	61 ± 3	17 ± 1	5 ± 1
	Median	13 ± 4	0 ± 0	0 ± 0
	95th %ile	253 ± 9	112 ± 3	32 ± 4
Urgent2	Mean	128 ± 8	27 ± 2	7 ± 1
	Median	21 ± 5	0 ± 0	0 ± 0
	95th %ile	591 ± 40	171 ± 8	45 ± 7
Urgent3	Mean	224 ± 37	35 ± 2	8 ± 2
	Median	32 ± 8	0 ± 0	0 ± 0
	95th %ile	1113 ± 194	220 ± 4	46 ± 18
Add-on Elect	Mean	340 ± 28	40 ± 3	10 ± 1
	Median	37 ± 10	0 ± 0	0 ± 0
	95th %ile	1745 ± 178	256 ± 18	55 ± 10
Utilization (%)		74.8 ± 0.5	55.9 ± 0.2	45.0 ± 0.3

Data are Mean ± SD. The n in parentheses aside number of ORs refers to the number of simulation runs performed

We then turn to a more complicated model in which we fix the number of ORs available during the day to 4 and the number of ORs at night to 2, 3 or 4. In addition, in this model nighttime surgery was restricted to emergency and significantly urgent patients (e.g., Urgent1 classification). With 4 ORs during daytime and at night, we found that wait times were short and within clinically acceptable ranges (Table 3). For example, the median wait time for emergency patients was 0 min, the mean was 14 min and the 95th percentile was 89 min. When the number of night time ORs was decreased wait times increased, as expected, especially in the higher urgency groups. For example, decreasing the number of ORs at night from 4 to 3 increased the wait times for emergency and urgent1 cases by 20–30 min at the 95th percentile (Table 3). When running just 2 ORs during the night, wait times for emergency cases averaged 29 min and the 95th percentile was at 144 min (although the median remained at 0 min; Table 3).

**Table 3:** Wait times (minutes) according to urgency classification and number of operating rooms running during the day and the number running at night

		Number of Operating Rooms		
		4, 4 (*n* = 4)	4, 3 (*n* = 4)	4, 2 (*n* = 4)
Emergent	Mean	14 ± 1	19 ± 1	29 ± 1
	Median	0 ± 0	0 ± 0	0 ± 0
	95th %ile	89 ± 1	109 ± 3	144 ± 3
Urgent1	Mean	19 ± 1	26 ± 1	44 ± 1
	Median	0 ± 0	0 ± 0	0 ± 0
	95th %ile	118 ± 3	149 ± 4	227 ± 9
Urgent2	Mean	128 ± 2	136 ± 1	138 ± 4
	Median	28 ± 2	34 ± 4	41 ± 2
	95th %ile	468 ± 5	474 ± 6	480 ± 7
Urgent3	Mean	148 ± 7	150 ± 16	159 ± 3
	Median	33 ± 7	34 ± 20	44 ± 6
	95th %ile	515 ± 23	559 ± 41	592 ± 20
Add-on Elect	Mean	176 ± 4	182 ± 11	194 ± 6
	Median	40 ± 8	39 ± 16	45 ± 3
	95th %ile	664 ± 18	692 ± 53	764 ± 57
Utilization (%)		55.7 ± 0.4	61.2 ± 0.5	66.9 ± 0.2

Data are Mean ± SD. The first number refers to the number of operating rooms running during daytime (0600–2200; 16 h) and the second number refers to the number of ORs running at night time (2200–0600; 8 h). The n in parentheses aside number of ORs refers to the number of simulation runs performed

Changing the clean-up time/surgical time affected wait times in a predictable way (Table 4). When clean-up/surgical time was decreased by 15 min, wait time for emergency cases decreased by 10 min for the 95th percentile, and decreased 20–65 min for urgent cases. Increasing the clean-up/surgical time by 15 min increased wait times, although the absolute change was greater than for the simulations with a 15 min decrease: at the 95th percentile, emergency cases waited 25 min longer, while for urgent classes, wait times increased 34–107 min.

**Table 4:** Effect of length of surgical time (or clean up time) on wait times (minutes) according to urgency classification

		4, 2, −15 min (*n* = 4)	4, 2 (*n* = 4)	4, 2, +15 min (*n* = 4)
Emergent	Mean	26 ± 1	29 ± 1	36 ± 1
	Median	0 ± 0	0 ± 0	0 ± 0
	95th %ile	134 ± 7	144 ± 3	169 ± 3
Urgent1	Mean	38 ± 1	44 ± 1	54 ± 2
	Median	0 ± 0	0 ± 0	0 ± 0
	95th %ile	205 ± 6	227 ± 9	261 ± 11
Urgent2	Mean	130 ± 2	138 ± 4	159 ± 5
	Median	29 ± 3	41 ± 2	63 ± 4
	95th %ile	466 ± 5	480 ± 7	537 ± 12
Add-on Elect	Mean	179 ± 2	194 ± 6	260 ± 6
	Median	38 ± 6	45 ± 3	92 ± 7
	95th %ile	665 ± 12	764 ± 57	1068 ± 39
Utilization (%)		63.1 ± 0.5	66.9 ± 0.2	70.9 ± 0.4

Data are Mean ± SD. The model assumes four operating rooms running during daytime (0600–2200; 16 h) and two ORs running at night time (2200–0600; 8 h). The model adds15 min (+15 min, right column) to (or subtracts15 min from, −15 min, left column) the length of the surgery. This 15 min change could also simulate 15 min of increased or decreased clean-up (turnover) time. The n in parentheses aside number of ORs refers to the number of simulation runs performed

Increasing patient volume increased wait times (Table 5). Increasing volume by 5 % increased wait time for urgent cases by 27–108 min at the 95th percentile; a 10 % volume increase resulted in an increase in 95th percentile wait times of 48–230 min for the urgent cases.

**Table 5:** Effect of surgical volume on wait times (minutes) according to urgency classification

		4, 2 (*n* = 4)	4, 2, +5 % (*n* = 4)	4, 2, +10 % (*n* = 4)
Emergent	Mean	29 ± 1	34 ± 1	39 ± 1
	Median	0 ± 0	0 ± 0	2 ± 1
	95th %ile	144 ± 3	157 ± 4	170 ± 5
Urgent1	Mean	44 ± 1	52 ± 1	59 ± 1
	Median	0 ± 0	0 ± 0	2 ± 1
	95th %ile	227 ± 9	254 ± 3	275 ± 6
Urgent2	Mean	138 ± 4	158 ± 7	173 ± 2
	Median	41 ± 2	62 ± 4	80 ± 4
	95th %ile	480 ± 7	534 ± 22	589 ± 11
Urgent3	Mean	159 ± 3	184 ± 11	222 ± 10
	Median	44 ± 6	58 ± 10	79 ± 12
	95th %ile	592 ± 20	705 ± 20	822 ± 27
Add-on Elect	Mean	194 ± 6	241 ± 26	301 ± 18
	Median	45 ± 3	81 ± 12	122 ± 8
	95th %ile	764 ± 57	937 ± 78	1233 ± 119
Utilization (%)		66.9 ± 0.2	70.2 ± 0.7	74.0 ± 0.1

Data are Mean ± SD. The model assumes four operating rooms running during daytime (0600–2200; 16 h) and two ORs running at night time (2200–0600; 8 h). The model adds patients (5 % increased volume, middle column; 10 % increased volume, right column). The n in parentheses aside number of ORs refers to the number of simulation runs performed

The mean wait times using a multiple server, multiple priorities waiting line model were similar to those obtained using Monte Carlo simulation (Table 6). In a four OR model, mean wait times between the two methods did not differ by more than 35 min, while the 3 OR model showed differences of 296 min for urgent3 patients. The use of log-transformed data to determine the mean surgical time resulted in better congruence between the Monte Carlo simulation and the standard approach, as compared to use of the mean of the raw data.

**Table 6:** Comparison of Monte Carlo simulation to standard approach. Waiting time (min)

4 ORs		Standard	Standard-Log	Monte Carlo
Emergent	Mean	16	9	11
Urgent1	Mean	23	12	15
Urgent2	Mean	38	18	23
Urgent3	Mean	61	26	34
Utilization (%)		55.9	47.9	54.7
3 ORs				
Emergent	Mean	53	32	37
Urgent1	Mean	87	48	54
Urgent2	Mean	195	91	114
Urgent3	Mean	462	166	226
Utilization (%)		74.5	63.9	73.3

The standard approach and Monte Carlo simulation used four (top) or three (bottom) operating rooms (ORs). Because the standard approach we used accepts a maximum of four urgency classes, we combined the 0–24 h urgency class with the add-on elective class for both Monte Carlo simulation and the standard approach. In the first column the surgical time was based on the mean of the actual surgical times of urgent cases for 1 year at our institution (plus 60 min preparation and clean-up time; total 244.76 min). The second column (Standard-Log) is based on a log transformation of the actual times (plus 60 min preparation and clean-up time; total 210 min). Note that the Monte Carlo simulation produced results closer to the log-transformed data. The standard approach produces mean values, but no variances because it is formulaic-based. The Monte Carlo data are from one simulation run, although the expected variation can be seen from the variation in the data of Table 2

Data generated using the “bootstrapping” method were similar to data using multiple 5 year simulations. For example, the median wait time for emergency patients differed by just 2 min (8 min versus 10 min) for 3 ORs and was 0 min for 4 and 5 ORs for both methods. Likewise, the difference between the two methods in the 95th percentile ranged from 1 to 4 min. As the urgency class became less acute, the difference widened. For example, differences in the 95th percentile ranged from 2 to 5 min for urgency1 patients to 4–139 min for add-on elective patients; the ranges of differences of the medians were 0–17 min.

**Fig. 3:**
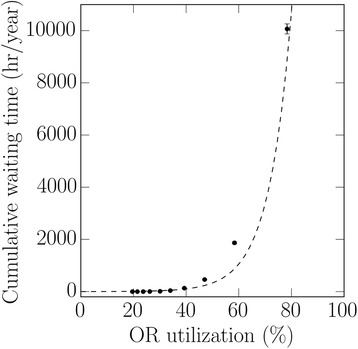
This graph shows the relationship between operating room (OR) utilization and waiting time. The simulation model was used to generate a large range of utilization scenarios; each scenario represents about 4 years of simulated data and the time represents the time (hours/year) patients had to wait. The number of ORs (range 3–12) was varied to achieve the different utilizations. Note that waiting time increased as the utilization increased, with an exponential rise at around 70–75 %. These data are consistent with the classical relationship between wait time and utilization. The error bars are the standard deviation; when error bars are not seen they are contained within the corresponding symbol

The effect of utilization on wait times is shown in Fig. 3. As expected, when parameters were altered to increase utilization (e.g., decreasing the number of available ORs), wait time increased, and did so exponentially when utilization approached 70–75 %.

## Discussion

The present study demonstrates a simulation approach to determine the resources needed to handle urgent surgical cases. We performed a sensitivity analysis and found how wait times change as the result of changing the number of ORs, the service time (e.g., how long resources are devoted to the patient) and surgical volume. The parameters of the program (which is freely available) can be adjusted according to the characteristics of individual hospitals. For example, the number of ORs needed to achieve acceptable wait times will depend on the arrival rate of patients, length of surgical procedures and preparation/clean-up time specific to each hospital.

In the present simulation model the arrival time equates to when the decision is made to perform surgery, and the wait time is the time between the arrival time and when the patient enters the OR. The interpretation of that wait time is made from a clinically relevant perspective, i.e., how long can the patient wait before a further delay would result in a clinically poorer outcome? But a patient might want to have surgery as soon as possible, even though waiting 24–48 h might not result in clinical compromise and therefore would be clinically acceptable. Thus, from a patient satisfaction perspective, a clinically acceptable wait time might not be acceptable to the patient.

We described our simulation data using mean, median and 95th percentile wait times, however, a manager could also determine the probability that a patient would need to wait a set time, such as 1 h or longer. For example, in the situation of running 3 ORs during daytime and at night, the probability that an emergency patient would wait 1 h or more is about 27 %.

The OR is one of the most resource-intensive parts of a hospital and so there is always a constant challenge to find the optimal balance between having enough ORs to provide timely peri-operative care and having the fewest number of ORs to minimize costs [10]. A fundamental issue that each hospital must address is the number of ORs that should be devoted to elective workflow and the number of ORs that should be reserved for non-elective patients (e.g., urgent and emergency patients). Some authors have recommended that during daytime weekday hours 15–20 % of available ORs be used for non-elective cases and last-minute elective cases. Depending on the demand for elective surgery, this approach could provide timely urgent and emergency surgical care at the expense of delayed scheduling of elective surgeries. This can have a negative influence on the programmatic visions and developments of the institution. Contrariwise, if all the ORs are used for elective care, patients needing urgent care will wait longer, which can increase emergency department boarding, wait times and the familiar problem of congestion.

Opening more ORs to accommodate urgent cases obviously comes at a financial cost. At our institution, the marginal cost to staff an OR 24 h/day, 365 days/year is about $1.3 million (United States dollars). This figure includes nurses and surgical technicians, but excludes anesthesia services and surgeon costs. In our model of staffing 4 ORs during the day and 2 ORs at night, opening up another OR at night (total 3 ORs at night, or an additional 8 h/day) would save around 860 h/year of patient waiting time at a cost of about $500/hour, or around $430,000/year. Going from 3 ORs to 4 ORs at night would result in a further reduction of patient waiting time by 550 h/year at a cost of $800/hour. Stated another way, because the marginal reductions in waiting time decrease as more ORs are staffed, but the marginal costs remain more-or-less the same, the cost per hour saved increases.

Queuing theory is used as part of a broad approach to smooth patient flow [11]. Patient flow issues have become an important area of focus by not only patients and healthcare workers, but also regulatory agencies, such as The Joint Commission. The ORs (and, by extension, the post-anesthesia care unit) are at the nexus of patient flow. Variability of patient flow in a hospital largely depends on variation of elective admissions, including surgical admissions. As part of the approach to reducing variability, some authors have recommended separating elective admissions from non-elective admissions, especially non-elective surgical patients [12, 13]. At Cincinnati Children’s Hospital and Mayo Clinic-Jacksonville, some ORs are dedicated to urgent cases [11]. This approach has helped reduce variability, and improved throughput and financial performance. The key concept is that the variability encountered in the ORs and hospital is categorized either as “natural (from the ED and needs to be managed) or “artificial” (from elective cases that need to be controlled).

We compared our simulation approach to a simplified queuing model which uses the mean duration of the surgical procedure (Table 6). There are several limitations of this latter approach. First, the model provides an average wait time, but does not provide a range of wait times. Thus, the mean wait time might seem acceptable but the wait times at the 95th percentile (e.g., for 5 % of patients) might be unacceptable. Secondly, using the average surgical time could be misleading if the surgical times are not normally distributed, as was the case for procedures at our institution. While this factor could be minimized by adding an *ad hoc* factor to account for the long tail of the duration of surgical procedures, our model can directly incorporate the observed distribution.

Several events must happen to ensure timely surgical care. The patient must be ready from a psychological and medical perspective; anesthesia, nursing and surgical staff must be available; and an OR must be open and ready to go. Patients cannot receive surgical care if any of these components is missing. Thus, this model assumes an alignment of resources, which clearly does not always occur. At our institution, surgeons are often available when the OR is not, and vice versa. Likewise, there may be limited nursing staff either because of unpredictable sick leave or boarding in the postoperative care unit that influences the ability to perform urgent cases in a timely fashion. There are numerous other patient flow variables that can impact patient wait times but use of our model provides a starting point for addressing them systematically. Traditionally, resources have been devoted to ensuring that the OR is always available, but such a model might no longer be economically viable, given the constraints on funding of healthcare. Thus, surgeons might need to alter their practice patterns to ensure better alignment of their availability with availability of the ORs. From the patient’s perspective, it matters little if the delay is due to lack of an OR or due to lack of a surgeon.

Wait time for surgery is a significant factor in the quality of care. First, the clinical condition of the patient can deteriorate during waiting, and is especially important for patients with emergency and urgent clinical disease. In particular, a patient who has traumatic injury and is hypovolemic and hypotensive requires immediate surgical care. Thus, waiting just a few minutes could be detrimental. Second, wait times negatively affect patient satisfaction [14, 15]. Third, excessive wait times can lead to increased costs [16]. Nonetheless, our data do not address the issue of what is “clinically acceptable waiting times”, although we have used that term. It is reasonable to argue that any patient who must wait beyond the established time has waited too long, yet a hospital might not want to devote resources to prevent such an occurrence. We found that a combination of 4 ORs during the daytime and evening and 2 ORs at night were sufficient, although more than 1 emergency patient in 20 would need to wait >2 h. It seems prudent that a call team could be used to mitigate such events, however, having 3 ORs at night might also be a reasonable approach. In addition, the 60 min “clean-up/preparation” time that we utilized can be significantly shortened in a real situation when a life-threatening emergency case arrives, or when a patient with less urgency has been waiting. Thus, our simulation program likely overestimated wait times at the 95th percentile for these cases. Last, at our institution, like at many other hospitals, patients are brought to a holding area near the ORs while the OR is being prepared. The patient can then enter the OR immediately when the OR is ready. For many of the scenarios that we modeled the median wait time was 0 min, which simply means that when the decision was made to perform surgery, an open OR was available and ready to accept the patient. We recognize, however, that it takes time to transport the patient to the OR.

Although it makes intuitive sense that reserving an OR for urgent cases should reduce waiting times and improve outcomes, studies have not uniformly shown positive benefits. Heng and Wright found that a dedicated OR for acute surgical cases at a children’s hospital reduced wait times by about 1 h, with a slight increase in patients who had surgery within 12 h (from 52 to 58 %) [17]. Trydestam et al. did not find that a dedicated OR improved timeliness of surgery for patients requiring laparoscopic cholecystectomies, appendectomies and repair of small bowel obstructions [18]. Likewise, using a simulation model, Wullink et al. did not observe benefits to a dedicated OR [19]. Others have reported increased delays and transfer of care, presumably because patients wait until the next day to have surgery in the dedicated OR [20]. Bhattacharyya and colleagues reported that an open OR for orthopedic cases decreased the proportion of hip fracture patients having surgery after 5pm; fewer complications occurred [21].

Cardoen et al. and others provide an extensive review of various methods and techniques related to OR scheduling [4–6]. A full comparison of these approaches is beyond the scope of the present paper. Cardoen et al., however, separate the methods into several broad categories, including mathematical programming, simulation and improvement heuristic [4]. It is important to note that these methods are not mutually exclusive: more than one can be applied to solve a particular scheduling problem. In addition, Pandit and colleagues have described methods to better manage surgical capacity and demand and thereby improve elective and urgent surgical utilization [22, 23].

## Conclusions

Our simulation program and approach provides a guide to determine how many ORs should be devoted to managing patients who require non-elective surgery. While we have tailored our approach based on the number of patients at our institution, the program can be adapted to predict resource needs at any institution, based on specific characteristics of each institution.

## Abbreviations

ORs: Operating rooms
UCDMC: University of California Davis Medical Center
